# Clinical validation of a novel web-application for remote assessment of distance visual acuity

**DOI:** 10.1038/s41433-021-01760-2

**Published:** 2021-08-30

**Authors:** Arun James Thirunavukarasu, Deborah Mullinger, Remi Mohan Rufus-Toye, Sarah Farrell, Louise E. Allen

**Affiliations:** 1 Department of Ophthalmology, Cambridge University Hospitals NHS Trust, Cambridge, UK; 2 School of Clinical Medicine, University of Cambridge, Cambridge, UK

**Keywords:** Diagnosis, Health services, Eye manifestations

## Abstract

**Background/Objectives:**

Ophthalmic disorders cause 8% of hospital clinic attendances, the highest of any specialty. The fundamental need for a distance visual acuity (VA) measurement constrains remote consultation. A web-application, DigiVis, facilitates self-assessment of VA using two internet-connected devices. This prospective validation study aimed to establish its accuracy, reliability, usability and acceptability.

**Subjects/Methods:**

In total, 120 patients aged 5–87 years (median = 27) self-tested their vision twice using DigiVis in addition to their standard clinical assessment. Eyes with VA worse than +0.80 logMAR were excluded. Accuracy and test-retest (TRT) variability were compared using Bland–Altman analysis and intraclass correlation coefficients (ICC). Patient feedback was analysed.

**Results:**

Bias between VA tests was insignificant at −0.001 (95% CI −0.017 to 0.015) logMAR. The upper limit of agreement (LOA) was 0.173 (95% CI 0.146 to 0.201) and the lower LOA −0.175 (95% CI −0.202 to −0.147) logMAR. The ICC was 0.818 (95% CI 0.748 to 0.869). DigiVis TRT mean bias was similarly insignificant, at 0.001 (95% CI −0.011 to 0.013) logMAR, the upper LOA was 0.124 (95% CI 0.103 to 0.144) and the lower LOA −0.121 (95% CI −0.142 to −0.101) logMAR. The ICC was 0.922 (95% CI 0.887 to 0.946). 95% of subjects were willing to use DigiVis to monitor vision at home.

**Conclusions:**

Self-tested distance VA using DigiVis is accurate, reliable and well accepted by patients. The app has potential to facilitate home monitoring, triage and remote consultation but widescale implementation will require integration with NHS databases and secure patient data storage.

## Introduction

Corrected distance visual acuity (VA) is a fundamental measure of visual resolution and is assessed before every ophthalmic, optometric, and orthoptic examination to inform clinical decision making. The standard method for VA assessment requires a trained observer to assess the smallest optotype that a patient can identify on an illuminated chart displayed at a set viewing distance. In specialist practice, the Snellen chart has largely been replaced by the Early Treatment of Diabetic Retinopathy Study (ETDRS) chart based on the logarithm of the Minimal Angle of Resolution (logMAR), conferring a more accurate estimation of VA than the Snellen chart due to its greater number of letter rows and the even crowding of letters on each line [1]. Variation in concentration, chart properties, viewing distance and observer bias are known to cause substantial fluctuations in test repeatability [2].

Ophthalmology clinics are the busiest in acute hospital trusts with nearly eight million attendances per year in the NHS; a high proportion are follow-up appointments for vision monitoring. This specialty workload is predicted to increase in coming years [3], and the NHS Long Term Plan requires a third of appointments to become virtual to increase clinic capacity [4]. COVID-19 has generated even greater urgency for change, with infection risk minimised by reducing clinic footfall and increasing the efficiency of emergency triage [5]. These targets can only be reached if patients can accurately self-test and monitor their visual function at home. Although many apps have been marketed to enable self-testing of vision, the majority test near vision only and have not been validated or CE marked. There remains an urgent requirement for a validated, accurate, and reliable method of self-testing distance VA [6–8].

DigiVis (www.digivis.org) is a recently developed CE marked web-application which facilitates self-testing of VA at home. It requires two internet-connected devices: one device (tablet or laptop) acts as a distant letter display screen and is paired with a handheld device (smartphone or tablet), which serves as the patient input device. An automated psychophysical staircase algorithm automatically changes the letter size on the distance tablet, based on patient input, enabling threshold VA to be calculated and stated in a range of different formats (Snellen notation, logMAR and cumulative number of letters). Consistent crowding of optotypes potentially gives DigiVis the reliability benefits of the standard ETDRS chart whilst its automated staircase and reversal algorithm removes observer bias and may improve precision.

This study aimed to assess the accuracy and reliability of DigiVis VA self-testing and to quantify its usability and acceptability in patients attending a hospital ophthalmology clinic appointment.

## Materials and methods

### DigiVis technology

The DigiVis test can be undertaken on a range of devices. A tablet, laptop or desktop screen displays the distance letter test chart and a smartphone or tablet is used as the patient input device. An animated instruction video in the application demonstrates the calibration steps. The distant test chart screen resolution calculation is undertaken by dragging virtual callipers on the distant test chart to match the size of a credit or store card held up against it. Patients can then choose to measure their 2 m viewing distance either with a tape measure or by using a novel automated distance calibration system within the application. This system asks patients to focus the second device’s camera on a graphic presented on the larger device screen, first at 30 cm (the length of an A4 sheet of paper) then approximately 2 m distance. The application compensates for discrepancy from 2 m by adjusting optotype sizing to adapt for distance viewing between 1.5 and 2.5 m if the patient is unable to measure distance manually. The test will not start if the viewing distance lies outside the 1.5–2.5 m range.

**Fig. 1:**
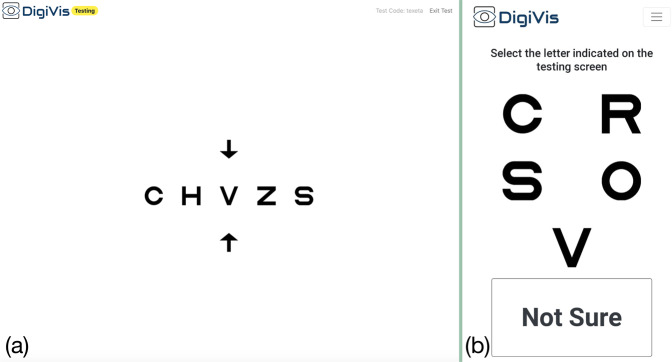
Screenshots from the DigiVis web-app during testing, from the larger screen (**a**) and handheld device (**b**). Patients select which optotype is indicated by arrows on the larger screen, from a range of choices on the handheld device.

Sloan letter optotypes are presented on the larger screen with crowding consistent with the letter size. The letter display is randomised and an arrow indicates the letter which the patient is required to identify (Fig. 1a). If able to recognise the letter, the patient selects the matching optotype out of a group of five displayed on their handheld device (four of which are randomised), or a ‘Not Sure’ option (Fig. 1b). For children under 10 years the test runs similarly, but features animations appearing upon letter selection, encouraging participation by gamifying the test: “collecting animals by matching letters”. Optotype size follows a modified García–Pérez psychophysical staircase with three reversal points, facilitating calculation of VA according to the smallest identifiable letter size [9]. A lower limit of 0.00 logMAR was set as a minimum threshold in this investigation to reduce testing time. The duration of the test may range from 30 s to 2 min, depending on the consistency of subjects’ responses.

### Participants

This was a prospective validation study comparing DigiVis VA self-testing to standard clinical testing. All procedures adhered to the tenets of the Declaration of Helsinki for research involving human subjects and the protocol was reviewed by the Health Research Authority and Health and Care Research Wales Ethics Committee.

Over a two-month period during the COVID-19 lockdown, research administrators posted study participation invitations and information leaflets to all patients due to attend a follow-up face to face eye clinic appointment. Inclusion criteria were as follows: age over four years, VA of +0.8 logMAR (6/38 Snellen) or better in each eye and no documentation of cognitive disability or requirement for interpreting services in the electronic patient records. The invitation letter encouraged patients who had access to two internet connected digital devices and wanted to participate to contact a clinician researcher. The clinician researcher phoned the patient to explain the study, take verbal consent and give password access to the DigiVis test.

### Testing

Patients were requested to undertake DigiVis testing twice before their clinic appointment, write down their VA scores and complete a usability and acceptance questionnaire to bring to the clinic with them. They were asked to contact the clinician researcher if they needed technical support during the tests. Written informed consent was given at the time of the hospital attendance.

Patients who had agreed to participate but forgotten to undertake the testing at home were re-invited to participate at the time of their clinic attendance and, after giving written informed consent, undertook DigiVis testing in a side-room at clinic. Patients were asked to watch the instruction video on the website and set up the system themselves, but medical students were on hand to offer technical support if required. Those using DigiVis at home used their own internet-connected devices. Those using DigiVis in clinic used their own handheld device with a department computer as the test chart. Background and device lighting were not controlled, and patients could modify their environment as desired.

At participants’ appointments, a standard, age-appropriate clinical assessment of VA was undertaken by nurse, optometrist, or orthoptist (blinded to previous DigiVis acuity measurement) and recorded. Patients were asked to hand in a usability and acceptance questionnaire alongside their consent form for DigiVis during their clinic visit. Paediatric patients completed a simpler feedback questionnaire and provided a form of written informed assent; parents or guardians completed the standard questionnaire and provided written informed consent.

### Analysis

Data from right eyes only were analysed, to avoid co-dependence. Agreement between DigiVis and clinical VA measurements, as well as test-retest (TRT) agreement was evaluated with Bland-Altman plots, looking specifically at 95% limits of agreement (LOA) and mean bias, and with mixed effects-model two-way intraclass correlation coefficients (ICC).

Where the standard assessment was undertaken using a Snellen chart, VA was converted to logMAR in Microsoft Excel [10] (v16.45, Microsoft Corporation, Redmond, Washington, United States). Analysis and data visualisation were conducted in R (v3.6.1, R Foundation for Statistical Computing, Vienna, Austria) and Affinity Designer (v1.8.6, Pantone LLC, Carlstadt, New Jersey, United States).

## Results

### Participant demographics

**Fig. 2:**
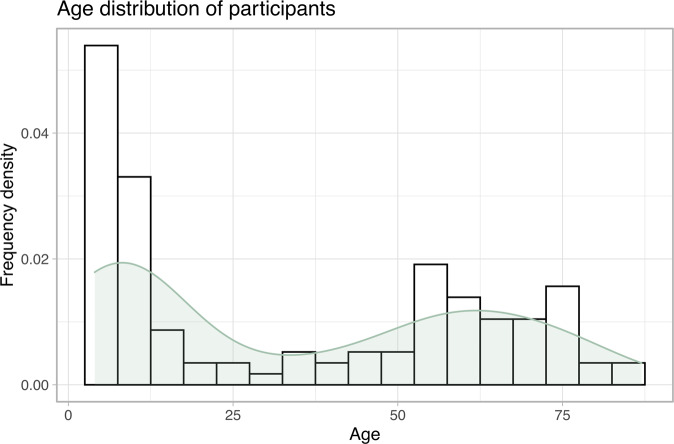
Histogram and overlain density plot illustrating the distribution of age in the participating population.

in total, 511 invitations to participate in the study were posted to eligible patients by the research team. The right eyes of the 120 patients who responded (23%) were tested using the DigiVis app and by standard, age-appropriate clinical assessment by a trained healthcare worker. Of these patients, 105 (88%) completed two DigiVis tests, enabling TRT agreement to be appraised. Subject VA based on standard clinical testing ranged from less than 0–0.8 logMAR (mean 0.07 logMAR). Patient ages ranged from 5–87 years, distributing as depicted in Fig. 2. There were 44 (37%) 5–10 year olds (instructed to undertake the children’s version of the DigiVis test), 32 (27%) between 55 and 75, and seven (6%) over 75. 36 (30%) patients undertook the DigiVis test in a side-room at clinic prior to their appointment; 84 (70%) undertook self-testing at home.

### Accuracy and reliability of visual acuity measurements

**Fig. 3:**
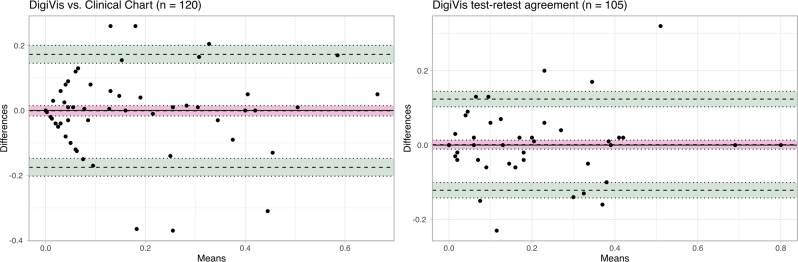
Bland-Altman plots comparing DigiVis and clinical measurements (right), and repeated DigiVis measurements (left). The bias and 95% LOA are indicated by dashed lines, whilst shaded 95% confidence intervals for each are bounded by dotted lines.

To facilitate Bland-Altman analysis, differences are plotted against mean values, for the first DigiVis VA and clinical measurement, as well as for repeated DigiVis VA measurements (Fig. 3). Bland-Altman bias and limits of agreement, as well as ICC and associated statistics, are stated in Table 1. In both cases, the mean bias was not statistically significantly different from 0.00 logMAR as indicated by the inclusion of the x-axis within the confidence intervals. LOA lie on average at ±0.174 logMAR when comparing DigiVis and clinical measurements, and at ±0.123 logMAR when comparing repeated DigiVis measurements. In both comparisons, there was no significant correlation (Pearson’s correlation coefficient, *p* > 0.05) between differences in VA and means of VA, indicating consistent performance across the tested range.

**Table 1:** Bland-Altman plots’ bias and 95% limits of agreement, and intraclass correlation coefficients with associated F value, degrees of freedom and *p* value, assessing agreement between DigiVis and clinical measurements, as well as between repeated DigiVis measurements of VA.

Comparison	*n*	Bias (95% CI)	Upper 95% limit of agreement (95% CI)	Lower 95% limit of agreement (95% CI)	Intraclass correlation coefficient (95% CI)	F_icc_ (df)	*p*_*icc*_
DigiVis-clinic	120	−0.001 (−0.017 to 0.015)	0.173 (0.146 to 0.201)	−0.175 (−0.202 to −0.147)	0.818 (0.748 to 0.869)	9.9 (119, 119.0)	2 × 10^−30^
Test-retest	105	0.001 (−0.011 to 0.013)	0.124 (0.103 to 0.144)	−0.121 (−0.142 to −0.101)	0.922 (0.887 to 0.946)	24.4 (104, 104.1)	9 × 10^−45^

ICCs indicated good (over 0.75) and excellent (over 0.90) agreement when comparing DigiVis and clinical measurements, and repeated DigiVis measurements, respectively. Similarly strong agreement was observed in left eyes.

### Patient feedback

Feedback was very positive: 49/53 (92%) children, 49/50 (98%) parents, and 57/61 (93%) adult patients rated DigiVis as’good’ or’excellent’, and 95% of patients and parents said they would be willing to use DigiVis to monitor vision at home. Patients were especially enthusiastic about the website’s detailed instructions, and option to potentially conduct a home test rather than attend a clinic. Of individuals that provided negative feedback, the most common issues were a lack of suitable devices to conduct the test at home, and concern that results may not be accurate.

## Discussion

Conventional chart-based assessment of VA has reported TRT LOA of ±0.15 logMAR [11], close to a theoretical maximum of ±0.14 logMAR determined in strictly controlled conditions [2]. Peek Acuity, a distance VA testing app, has reported LOA between app and clinical measurements of ±0.444 logMAR and TRT LOA of ±0.414 logMAR [12]. The LOA between the Kays pictures iSight app and clinical measurements is reported as ±0.125–0.208 logMAR [13]. COMPlog, a distance VA test requiring a specifically sized computer monitor and trained observer, recorded TRT LOA at ±0.10–0.12 logMAR [14], and ICC of 0.964 when comparing face to face with remote testing [15]. Other smartphone-based VA testing apps have reported LOAs of ±0.198 logMAR [16] and ±0.10-0.30 logMAR under clinical supervision [17]. Together, these data provide a priori standards by which DigiVis can be evaluated.

In this study, DigiVis self-test VA assessment had minimal bias, LOAs of ±0.174 when compared to standard clinical testing, and TRT LOAs of ±0.123 logMAR, without clinical supervision, favourable compared to other apps, and even clinical testing. High ICC values reinforce evidence of its accuracy and reliability. The narrowness of confidence intervals for calculated statistics suggests that the sampled population was sufficiently large to provide robust results. Advantages of DigiVis include the automated calculation and calibration of distance between patient and screen (overcoming the known issue of observer distance determination affecting test results), as well as the use of crowded letter optotypes which enable direct comparison to previous standard clinical testing. The forced-choice nature of the DigiVis test and the absence of observer bias are likely to result in improved test reliability relative to clinical assessment [18].

There were several limitations of this study. The number of participants was smaller than the number invited; a proportion of those that did not participate may not have had digital access to the test. Although 70% of DigiVis testing was conducted successfully at home on patients’ own devices, 30% undertook the test at clinic, which may have made gaining access and setting up the test easier. The age distribution of the sampled population was weighted towards the under-10 and 55-75 age groups. The relatively lower proportion of elderly participants likely reflects the pattern of attendance at eye clinics during the COVID-19 lockdown period. Standard clinical testing was carried out using a variety of standard charts: Snellen, ETDRS and children’s logMAR flip charts. This reflects real-world variation in ophthalmology clinics but may have reduced the reliability of clinical measurements. Another limitation of this analysis was the exclusion of patients with VA worse than +0.8 logMAR, a decision made due to the presumed difficulties these individuals may have in accessing the test. Few participants in this study had vision worse than 0.5 logMAR–further investigation in patients with poorer visual acuities is required to verify the app’s potential in this population. Finally, although DigiVis test-retest agreement may suggest superior reliability to clinical assessment, its apparent consistency may have been inflated by participants repeating the test in quick succession, in the same testing environment and on the same devices.

DigiVis represents a validated means of obtaining accurate and reliable visual acuity data without supervision. Particular benefits of the application include: (1) the nature of a distance VA test directly comparable to chart-based assessment; (2) complete automation of the test, not requiring clinician input; (3) CE marking and validated accuracy and reliability, and (4) measurement of viewing distance with compensatory size-adjustment of optotypes. This could prove useful in enabling patients to self-monitor and report their VA, augmenting remote consultations. Asynchronous (maximising patient autonomy) or synchronous (facilitating clinician input via screen-sharing) testing may be utilised as required. For those shielding or self-isolating for COVID-19, DigiVis VA assessment may enable a clinician and the patient to balance the urgency of an in-person consultation with the risk of hospital attendance. As a result of school closures, a year’s cohort of children have missed school vision screening (700,000 children in the UK). DigiVis assessment at home may facilitate catch-up vision screening, although further testing in 4 to 5-year-old children is required to confirm this. Individuals having difficulty in accessing the test due to the unavailability of digital devices, internet connection, or difficulties with the instructions could undertake testing in local community centres or be guided in its use through the screen-share functionality of video-consulting software.

This clinical validation study indicates that DigiVis self-testing is a viable alternative to standard clinical assessment of VA in patients aged five years and older. Further studies are needed to validate its use in the elderly, patients with VA worse than 0.5 logMAR, and vision screening in young children. The web-app has the potential to support triage and remote consultations for ophthalmology services and be used for VA testing in primary and secondary care where a standard chart or suitable trained examiner is not available, for instance, general practice, emergency departments, and in-patient wards. Additional support or alternative testing will still be necessary for patients with severe visual impairment, cognitive impairment, or who struggle with digital access. For selected patients, VA apps have the potential to reduce the need for clinic attendance, and workload, but require integration into patient pathways, clinician workflows, and electronic patient records.
